# Murder or Not? Cold Temperature Makes Criminals Appear to Be Cold-Blooded and Warm Temperature to Be Hot-Headed

**DOI:** 10.1371/journal.pone.0096231

**Published:** 2014-04-30

**Authors:** Christine Gockel, Peter M. Kolb, Lioba Werth

**Affiliations:** 1 Department of Psychology, University of Fribourg, Fribourg, Switzerland; 2 Building Physics Department, University of Stuttgart, Stuttgart, Germany; 3 Business and Organizational Psychology, University of Hohenheim, Stuttgart, Germany; University of Bologna, Italy

## Abstract

Temperature-related words such as cold-blooded and hot-headed can be used to describe criminal behavior. Words associated with coldness describe premeditated behavior and words associated with heat describe impulsive behavior. Building on recent research about the close interplay between physical and interpersonal coldness and warmth, we examined in a lab experiment how ambient temperature within a comfort zone influences judgments of criminals. Participants in rooms with low temperature regarded criminals to be more cold-blooded than participants in rooms with high temperature. Specifically, they were more likely to attribute premeditated crimes, ascribed crimes resulting in higher degrees of penalty, and attributed more murders to criminals. Likewise, participants in rooms with high temperature regarded criminals to be more hot-headed than participants in rooms with low temperature: They were more likely to attribute impulsive crimes. Results imply that cognitive representations of temperature are closely related to representations of criminal behavior and attributions of intent.

## Introduction

In July 2011, Anders Behring Breivik set off a car bomb in Oslo killing eight people. Two hours later, he opened fire at the participants of a nearby summer camp killing 69 people and injuring more than a hundred people. In the media, his meticulously planned behavior was described as cold-blooded, cold-hearted, and ice-cold. This choice of words demonstrates that we use characteristics from our physical environment (such as cold) to describe human behavior and characteristics. It exemplifies how strongly our physical and mental worlds are interconnected.

Mental concepts about the environment or other people (e.g., cold-blooded, warm-hearted, or hot-headed behavior) are grounded in direct and concrete physical experiences [1], [2], [3]. For example, caretakers who hold a baby closely and provide a warm hug might provide love and support (i.e., psychological warmth) at the same time. Likewise, not holding a baby closely and rather keeping the baby at a distance might signal lack of love and support (i.e., psychological coldness). This tight connection between physical and social experiences can help explain why we so easily use metaphors based on physical experiences when referring to more abstract social concepts [4].

Research in the past few years has examined this tight connection and demonstrated how the sensation of physical temperature can affect judgment and behavior [5], [6], [7], [8]. Even the experience of holding a cup of cold or hot coffee impacts the assessment of a target person: Participants with iced coffee in their hand assessed a target person to be interpersonally colder than participants with warm coffee [7]. Thus, interpersonal feelings of coldness are linked to physical experiences. The main reason for this link, Williams and Bargh propose, is the simultaneous activation of the respective concepts “coldness” or “warmth”. Concepts related to physical and psychological coldness or warmth are also processed in the same area of the brain [9]. Likewise, hot temperature can affect psychological processes as well. In this case, the cognitive representations of heat and the negative emotion of anger are closely related. Wilkowski and colleagues [10] examined this link in several studies and showed, for example, that anger-related facial expressions were categorized faster when displayed on a background associated with heat (i.e., a fire). Their results suggest that triggering the concept heat activates anger associations.

In the case of Behring Breivik, temperature-related words were used to describe deviant or criminal behavior. His actions were described with various words indicating different degrees of cold-blooded behavior. Words associated with coldness typically indicate that the person acted with forethought. Likewise, a person or action can be described with various words indicating different degrees of hot-headed behavior, for example hot-tempered, fiery, and boiling. Words associated with heat typically indicate that the person acted spontaneously and impulsively – in the heat of passion [11]. Similar temperature-related terms with the same associations can be found in other languages, e.g. “leng xue – re xue” in Mandarin or “kaltblütig – hitzköpfig” in German. The distinction between cold/deliberate and hot/impulsive behavior is reflected in the definition of aggression as either cold/instrumental or hot/hostile aggression [12] and in the description of the cool and hot system of self-regulation [13]. It is also relevant in jurisdiction because cold-blooded behavior is associated with premeditated crimes and hot-headed behavior with impulsive crimes. Judges and jurors need to assess to what extent a defendant acted premeditatedly or impulsively [14], and this evaluation is a central determinant of the sentence.

All of these findings suggest that temperature should impact the judgment of criminals, more specifically the attribution of criminal intent and associated judgments. Because the experience of physical temperature activates psychological concepts associated with temperature [4], [5], [7], criminals should be considered as more cold-blooded in cold temperature. Thus, participants in cold temperature conditions, as compared to participants in medium or warm temperature conditions, should be more likely to attribute premeditated crimes and to attribute crimes associated with higher prison sentences. They should also be more likely to spontaneously attribute murder as crime because murder is commonly considered to be a cold-blooded crime. For a killing to be labeled as murder, premeditation or malice aforethought are required. According to German Criminal Code (Strafgesetzbuch), several qualifications are necessary for a killing to be labeled as murder (Mord). If these qualifications are not met, the killing is labeled as Totschlag (literally “deathblow”; similar to second-degree murder in the US). Killing someone on impulse is generally not considered to be Mord, but Totschlag. Murder is also associated with the highest possible prison sentences in most countries. Accordingly, we assume that the effects on criminality inferences are driven by low temperature.

Due to the association between high temperature and anger [10], though, criminals should be considered as more hot-headed in warm temperature. Thus, participants in warm temperature conditions, as compared to participants in cold or medium temperature conditions, should be more likely to attribute impulsive crimes. Accordingly, we assume that the effect on ratings of impulsivity is driven by high temperature.

In our research, we focus on a novel area of investigation, namely criminality inferences and attribution of criminal intent. To test our hypotheses, we conducted a lab experiment with student participants and manipulated ambient temperature. Previous research had shown that temperature can lead to changes in affect [15], which in turn influences cognitive processes such as interpersonal judgments [16], [17]. We therefore attempted to rule out that our effects were driven by affect by assessing and controlling for it.

## Method

### Ethics Statement

Data collection took place at Chemnitz University of Technology, Germany, in accordance with the ethical guidelines of the American Psychological Association (APA). Data were collected anonymously. At the beginning of the study, participants were explained the procedure of the study, had the opportunity to ask questions, and provided verbal consent. They were free to terminate the study at any point in time without any negative consequences.

### Design and Participants

The design of the study was a between-subjects single-factor design (ambient temperature: low vs. medium vs. high). Ambient temperature in the Low Temperature (Low-Temp) condition was 19.9°C (67.8°F), in the Medium Temperature (Mid-Temp) condition 23.8°C (74.8° F), and in the High Temperature (High-Temp) condition 26.2°C (79.2° F). These temperatures were chosen because they correspond to the lower, medium, and upper levels of the comfort zone. The comfort zone's limits for indoor environments are approximately 20°C (68°F) and 28°C (82.4°F; ASHRAE, 2010). To calculate operative ambient temperature according to ISO 7726:1998 (International Organization for Standardization, 1998), air temperature, globe temperature and air velocity were measured.

One hundred and forty-seven students from Chemnitz University of Technology in Germany were recruited via the psychology department participant pool and university mailing lists. The sample size was determined before the study by running a power analysis assuming that differences between conditions would be of medium size. Participants received either research credit or €5 ($6.60) in cash for their participation and were assigned randomly to one of the three conditions. A total of fourteen participants (9.5%) had to be dropped from the analyses for the following reasons: eight participants because the air conditioner or fan heater malfunctioned, two participants due to extreme temperature fluctuations during the experimental session, three participants because their temperature evaluations qualified as outliers (and were either 3 SD above or below the mean), and one participant because of not answering the relevant questions. Thus, 133 participants remained in the analyses (79% female, *M*_age_ = 22.31 years, *SD* = 3.39). They were evenly distributed among conditions (Low-Temp: *n* = 44; Mid-Temp: *n* = 45; High-Temp: *n* = 44).

### Procedure and Measures

To manipulate ambient temperature, a mobile air conditioner or a fan heater, hidden behind a separating wall, were employed. The laboratory had three workplaces equipped with conventional PCs allowing for simultaneous data collection of three participants. When arriving at the lab, the experimenter told participants that the focus of the study was person perception and that several measurements within the room were being conducted during the session. First, participants worked on unrelated tasks for around 15 minutes to acclimatize to the temperature. Then they filled out a questionnaire on affect and worked on the experimental task.

#### Affect questionnaire

Both negative and positive affect were assessed with items taken from the German language adjective list EWL [18]. For negative affect, participants indicated, on 7-point Likert-type scales from 1 (*strongly disagree*) to 7 (*strongly agree*), to what extent they felt unhappy, sad, discouraged, and sorrowful. Cronbach's α for negative affect was .91. For positive affect, participants indicated on the same Likert-type scales to what extent they were glad, cheerful, and in a good mood. Cronbach's α for positive affect was .90.

#### Experimental Task

To assess judgments of criminals, eight photos (four females and four males) were presented to participants in randomized order. They were taken from an online database of mugshots [19], which displays pictures of people arrested and photographed. Participants read that photos showed persons who had committed a crime. Then they were asked to spontaneously attribute a crime to each criminal by answering the question “What kind of crime did this person commit?” via an open response format.

#### Degree of penalty

After data collection, two independent raters, who were blind to conditions, assessed participants' answers. They ascribed a prison sentence to each attributed crime based on their lay understanding of German law. Ratings were conducted on a scale ranging from 0 (*The person is innocent*) to 180 months/15 years (*The person has committed a serious crime and received the maximum prison sentence*). 15 years is the maximum prison sentence according to German law and if no exceptional gravity of guilt is determined. Examples for crimes resulting in low prison sentences were theft, drug possession, and tax evasion and for crimes resulting in high prison sentences child assault, kidnapping, and murder. Agreement between raters was *r* = .93.

#### Attribution of murder

The two raters also counted how many murders each participant had attributed to the eight criminals.

#### Likelihood of premeditated and impulsive crimes

Participants were presented with all photos a second time and asked how likely each criminal had committed a premeditated crime and how likely each criminal had committed an impulsive crime. Both questions were answered on 7-point rating scales ranging from 1 (*very unlikely*) to 7 (*very likely*). By averaging ratings on all criminals, one score for the likelihood of premeditated crimes and one score for the likelihood of impulsive crimes were calculated.

#### Potential covariates

When investigating temperature effects, the following variables influencing temperature sensation should be considered: exposure duration, age, sex, outdoor temperature, and clothing [20], [21], [22], [23]. Exposure duration was participants' time to complete the experiment. Participants' clothing was rated by the experimenter on a visual scale from 1 (*summer attire*) to 8 (*winter attire*) [6].

#### Manipulation check

At the end of the session, participants estimated the laboratory's ambient temperature and indicated, on 7-point Likert-type scales from 1 (*strongly disagree*) to 7 (*strongly agree*), their agreement with the following two statements: It is too cold in this room. And, it is too warm in this room. After that, participants were thanked and debriefed.

## Results

### Preliminary Analyses

We first examined how strongly the potential covariates exposure duration, age, sex, outdoor temperature, and clothing correlated with all dependent variables of interest by analyzing bivariate correlations. No covariate correlated significantly with a dependent variable (all *r*'s<.13, all *p*'s>.07). Thus, the potential covariates were not used in the analyses.

### Manipulation Check

To find out whether the manipulation of ambient temperature had affected participants' temperature perception, we ran a MANOVA. Ambient temperature condition served as independent variable. The three manipulation check items temperature estimation, rating of the room as too cold, and rating of the room as too warm served as dependent variables. Results indicated that the manipulation had been successful because it had affected participants' temperature perception, Pillai's trace = .89, *F*(6,258) = 34.58, *p*<.001.

To find out about the specific effects of the ambient temperature manipulation on each manipulation check item, we subsequently ran three univariate ANOVAs. Condition means, standard deviations, and ANOVA results are displayed in Table 1. Results show that the manipulation had affected participants' answers on all three manipulation check items. We then used simple contrasts to compare the Low-Temp and High-Temp condition with the Mid-Temp condition, respectively. Contrasts showed that participants in the Low-Temp condition estimated the temperature to be lower than participants in the Mid-Temp condition (*p*<.001). They also showed more agreement with the statement that the room was too cold (*p*<.001) and less agreement with the statement that the room was too warm (*p* = .001). Participants in the High-Temp condition, in turn, estimated the temperature to be higher than participants in the Mid-Tem condition (*p*<.001). They showed more agreement with the statement that the room was too warm (*p*<.001) and the same amount of agreement with the statement that the room was too cold (*p* = .23).

**Table 1:** Means, Standard Deviations, and Overall ANOVA Results for all Variables.

		Temperature								
		Low		Medium		High		*F*(2, 130)		
Variable		*M*	*SD*	*M*	*SD*	*M*	*SD*		*p*	η^2^ *_p_*
1	Ambient temperature^a^	19.88	0.38	23.82	0.33	26.23	1.42	600.41	<.001	.90
2	Estimation of ambient temperature^b^	17.73	2.55	21.12	1.60	23.82	2.38	83.90	<.001	.56
3	“It is too cold in this room.”	5.32	2.04	1.87	1.12	1.50	0.93	93.66	<.001	.59
4	“It is too warm in this room.”	1.48	0.82	2.56	1.62	4.77	1.94	52.83	<.001	.45
5	Likelihood of premeditated crimes^c^	4.65	0.87	4.33	0.64	4.01	0.70	8.38	<.001	.11
6	Degree of penalty^d^	59.65	20.09	52.49	16.25	48.21	17.46	4.54	.012	.07
7	Attribution of murder^e^	0.77	0.94	0.38	0.68	0.36	0.78	3.66	.028	.05
8	Likelihood of impulsive crimes^c^	4.04	0.99	4.27	0.88	4.49	0.82	2.79	.065	.04
9	Negative affect	2.24	1.26	2.51	1.38	2.42	1.15	0.52	.599	.01
10	Positive affect	4.56	1.15	4.40	1.36	4.29	1.05	0.58	.562	.01

Note. *N* = 133.

a in degrees Celsius; operative ambient temperature was calculated according to ISO 7726:1998 (International Organization for Standardization, 1998) [26].

b in degrees Celsius.

c 1 (*very unlikely*) - 7 (*very likely*).

d in months; rated by two independent raters on the basis of attributed crimes.

e number of attributed murders to eight criminals.

### Main Analyses

Correlations between all study variables are displayed in Table 2. To test whether ambient temperature had affected criminality inferences, attributions of criminal intent, and affect, we ran a second MANOVA. Again, ambient temperature condition served as independent variable. All variables assessed in the experimental task and on the affect questionnaire served as dependent variables. Results indicated that the manipulation had affected a combination of the dependent variables, Pillai's trace = .18, *F*(12,252) = 2.08, *p* = .02.

**Table 2:** Zero-Order Correlations for all Variables.

		Intercorrelations						
Variable		1	2	3	4	5	6	7
1	Ambient temperature	―						
2	Estimation of ambient temperature	.71*	―					
3	Likelihood of premeditated crimes	−.29*	−.20*	―				
4	Degree of penalty	−.25*	−.08	.27*	―			
5	Attribution of murder	−.22*	−.03	.29*	.80*	―		
6	Likelihood of impulsive crimes	.18*	.14	−.35*	−.16	−.12	―	
7	Negative affect	.07	.10	.12	−.04	−.04	.06	―
8	Positive affect	−.06	−.04	.02	.14	.05	.02	−.54*

Note. *N* = 133.

*p<0.05.

To find out about the specific effects of the ambient temperature manipulation on each dependent variable, we subsequently ran univariate ANOVAs. Condition means, standard deviations, and ANOVA results are displayed in Table 1 as well.

First, the attribution of premeditated crimes differed significantly between conditions. We used simple contrasts to compare the Low-Temp and High-Temp condition with the Mid-Temp condition, respectively. Contrasts showed that participants in the Low-Temp condition were more likely to attribute premeditated crimes to presented criminals than participants in the Mid-Temp condition, *p* = .*04*. These, in turn, were more likely to attribute premeditated crimes to presented criminals than participants in the High-Temp condition, *p* = .04.

Second, degree of penalty (as rated by observers) differed significantly between conditions. Contrasts showed that participants in the Low-Temp condition tended to attribute crimes resulting in higher degrees of penalty than participants in the Mid-Temp condition, *p* = .06. But these, in turn, did not differ significantly from participants in the High-Temp condition, *p* = .26. Attributed crimes in the Low-Temp condition resulted in prison sentences of 59.65 months (i.e. almost five years) and in the other two conditions in 50.35 months (i.e. four years, 2 months), on average.

Third, the attribution of murder as crime differed significantly between conditions. Contrasts showed that participants in the Low-Temp condition attributed more murders to criminals than participants in the Mid-Temp condition, *p* = .02. These, in turn, did not differ significantly from participants in the High-Temp condition, *p* = .93. On average, participants in the Low-Temp condition attributed murder to 9.6% of the presented criminals (.77 out of 8). In the other two conditions, participants attributed murder to only 4.6% of the criminals (.37 out of 8). Thus, the probability of a spontaneous murder attribution was around twice as high in the Low-Temp condition than in the other two conditions. To sum, participants in low temperature were more likely to attribute premeditated crimes, tended to ascribe crimes resulting in higher degrees of penalty, and attributed more murders than participants in medium or high temperature. Thus, the previous effects seem to be driven more by low than by high temperature.

Our fourth outcome of interest were attributions of impulsive crimes: They tended to differ between conditions. Contrasts showed that participants in the Low-Temp condition did not differ significantly from participants in the Mid-Temp condition, *p* = .23, and that participants in the Mid-Temp condition did not differ significantly from participants in the High-Temp condition, *p* = .25. An exploratory follow-up t-test showed that participants in the Low-Temp condition differed from participants in the High-Temp condition, though, *t*(86) = 2.34, *p* = .02. Thus, the effect on attributions of impulsive crimes is not driven more by low temperature like the effects above.

Finally, we examined to what extent the ambient temperature manipulation had affected participants' affect. Neither positive affect nor negative affect were influenced by the temperature manipulation (see Table 1). Also, none of the results above could be explained by changes in negative or positive affect: All temperature effects on criminality inferences and attributions of criminal intent remained significant when negative and positive affect were added as covariates into the analyses.

## Discussion

In this study, we demonstrated that ambient temperature affects the judgment of criminals. Participants in rooms with low temperature were more likely to attribute premeditated crimes, tended to ascribe crimes resulting in higher degrees of penalty, and attributed more murders to criminals than participants in rooms with medium/high temperature. Likewise, participants in rooms with high temperature were more likely to attribute impulsive crimes than participants in rooms with low temperature. Findings could not be explained by changes in negative or positive affect. We base our reasoning on recent research examining the close interplay between physical and interpersonal coldness and warmth. Descriptions of cold-hearted and hot-headed behavior are more than simple expressions. They point to the fact that cognitive representations of temperature are closely related to representations of criminal behavior in general and of criminal intent more specifically.

Crucially, we could show that most effects are driven by cold temperature because we employed a control condition with medium temperature. Because we also measured negative and positive affect, we can rule out that effects are based on affective processes. Furthermore, we manipulated ambient temperature within a zone of thermal comfort, which is representative of all settings where criminals are likely to be evaluated (police buildings and courtrooms). Our study therefore proposes legal implications of priming effects and contributes to the literature due to its practical significance. However, one important limitation of this study should be noted: We did not measure directly which cognitive concepts were activated by ambient temperature. Hence, we are not able to explain the underlying process leading from the experience of ambient temperature to the assessment of criminals. In this regard, previous research has shown that feelings of loneliness and the need for affiliation can drive temperature effects [4], [6]. In our study, however, these processes do not seem to be theoretically relevant, because criminals should not be associated with satisfying one's need for affiliation. We assume instead that cold temperature might have activated concepts such as “cold” and “cold-blooded” leading to attributions of premeditated and more severe crimes and that warm temperature might have activated concepts such as “hot-headed” and “warm-hearted” leading to attributions of impulsive crimes. The latter two concepts might have been activated simultaneously, though, and resulted in a relatively weak overall effect because they partly counteract each other.

Our research complements earlier research on temperature-driven person perception by Williams and Bargh [7] because similar to these authors, we also show that colder temperatures lead to a more negative, i.e., colder, evaluation of target persons. Future research may focus on several issues that still remain unanswered: First, what are the boundary conditions for the effects? The role of temperature effects on the assessment of criminals might diminish as the availability of evidence increases. Temperature effects should be greatest under conditions of uncertainty and if evaluators lack information about a suspect's culpability. Second, how does the effect change in more extreme ambient temperatures? Following research on aggression, we assume that the relationship between temperature and attribution is not linear. Heat can impact aggression and may, at extreme levels, even lead to an increased willingness to escape from the unpleasant environment [24], [25]. This may fundamentally affect psychological processes like attribution as well. Third, does the effect extend to other targets and to behaviors? In courtrooms, temperature may also affect judges' and jurors' perceptions of other important actors like witnesses. In addition to perception and judgment, temperature can impact behavior [6], [7]. It may therefore also influence a suspect's behavior when being interrogated. This complex interplay of temperature effects on different actors needs to be investigated further.

To conclude, we demonstrated that temperature can affect attributional processes. In general, temperature effects should be especially strong when the behavior of others is evaluated in ambiguous situations. With this study, we have added one more important piece to the rapidly growing picture about the close interplay between physical and interpersonal coldness and warmth.
